# ALS-like pathology diminishes swelling of spinal astrocytes in the SOD1 animal model

**DOI:** 10.3389/fncel.2024.1472374

**Published:** 2024-10-10

**Authors:** Tereza Filipi, Jana Tureckova, Ondrej Vanatko, Martina Chmelova, Monika Kubiskova, Natalia Sirotova, Stanislava Matejkova, Lydia Vargova, Miroslava Anderova

**Affiliations:** ^1^Department of Cellular Neurophysiology, Institute of Experimental Medicine, Czech Academy of Sciences, Prague, Czechia; ^2^Second Faculty of Medicine, Charles University, Prague, Czechia; ^3^Faculty of Science, Charles University, Prague, Czechia; ^4^Analytical Laboratory, Institute of Organic Chemistry and Biochemistry, Czech Academy of Sciences, Prague, Czechia

**Keywords:** amyotrophic lateral sclerosis, SOD1, astrocytes, volume regulation, extracellular space, potassium uptake

## Abstract

Astrocytes are crucial for the functioning of the nervous system as they maintain the ion homeostasis via volume regulation. Pathological states, such as amyotrophic lateral sclerosis (ALS), affect astrocytes and might even cause a loss of such functions. In this study, we examined astrocytic swelling/volume recovery in both the brain and spinal cord of the SOD1 animal model to determine the level of their impairment caused by the ALS-like pathology. Astrocyte volume changes were measured in acute brain or spinal cord slices during and after exposure to hyperkalemia. We then compared the results with alterations of extracellular space (ECS) diffusion parameters, morphological changes, expression of the Kir4.1 channel and the potassium concentration measured in the cerebrospinal fluid, to further disclose the link between potassium and astrocytes in the ALS-like pathology. Morphological analysis revealed astrogliosis in both the motor cortex and the ventral horns of the SOD1 spinal cord. The activated morphology of SOD1 spinal astrocytes was associated with the results from volume measurements, which showed decreased swelling of these cells during hyperkalemia. Furthermore, we observed lower shrinkage of ECS in the SOD1 spinal ventral horns. Immunohistochemical analysis then confirmed decreased expression of the Kir4.1 channel in the SOD1 spinal cord, which corresponded with the diminished volume regulation. Despite astrogliosis, cortical astrocytes in SOD1 mice did not show alterations in swelling nor changes in Kir4.1 expression, and we did not identify significant changes in ECS parameters. Moreover, the potassium level in the cerebrospinal fluid did not deviate from the physiological concentration. The results we obtained thus suggest that ALS-like pathology causes impaired potassium uptake associated with Kir4.1 downregulation in the spinal astrocytes, but based on our data from the cortex, the functional impairment seems to be independent of the morphological state.

## 1 Introduction

Amyotrophic lateral sclerosis (ALS) is a fatal disease characterized by motor neuron (MN) degeneration, resulting in muscle atrophy. The atrophy severely lowers the quality of life and culminates in early death, with a median survival of three to 5 years from diagnosis. There is currently no cure or prevention available, mainly because the pathological mechanisms of the disease are incompletely understood.

Motor neurons were for a long time considered the only cell type affected by the pathology, but numerous studies have discovered that non-neuronal cells, such as glia, undergo changes and play a role in ALS progression too. Astrocytes were reported to have changed morphology and to lose neuro-supportive functions in the mutated superoxide dismutase (mSOD1) mouse – a model of ALS. The ablation of mSOD1 in astrocytes then caused significantly slower disease progression, emphasizing the active role of astrocytes in MN degeneration (Yamanaka et al., 2008).

Changes in astrocyte gene expression have been described in the context of ALS, and several mechanisms have been proposed for the presumed astrocytic contribution to MN death. They include glutamate excitotoxicity and ionic imbalance due to impaired astrocytic clearance. Physiologically, maintaining ion homeostasis is one of the most important functions of astrocytes in the CNS. They sustain the K^+^ concentration in the extracellular space (ECS) on a physiological level (around 3 mmoL/L) (Larsen et al., 2016) employing both ion channels (primarily inwardly rectifying K^+^ channels; Kir) pumps and transporters (Na^+^/K^+^-ATPase; NKA and Na^+^/K^+^/2Cl^−^ cotransporter; NKCC), which are necessary to prevent neuronal hyperexcitability. During the various pathological conditions, excessive release of K^+^ from neurons occurs, and due to energy deprivation and failure of astrocytic NKA, its concentration can reach up to 50–60 mM in case of ischemia or traumatic injury (van Putten et al., 2021). Disruption of K^+^ homeostasis results in increased activity of potassium channels and activation of NKCC1, which drives the import of Na^+^, K^+^, and Cl^−^, and results in extensive swelling of astrocytes and shrinkage of the CNS ECS (Hellas and Andrew, 2021; Kahle et al., 2015; Song and Yu, 2014). This can cause additional ion imbalance, affect neuronal excitability and synaptic transmission, and disrupt the movement of molecules and ions critical for nutrition delivery and signaling. That in turn leads to increased tissue density, cellular stress responses including inflammation and oxidative stress, and eventually results in cell damage or death (Amlerova et al., 2024; Nedergaard and Verkhratsky, 2012). Changes in ECS volume can be characterized by ECS diffusion parameters, extracellular volume fraction *α* and tortuosity *λ*, which reflect the contemporary structure of the brain and govern the extracellular diffusion of neuroactive substances (Thorne and Nicholson, 2006; Nicholson and Hrabetova, 2017).

Considering the importance of ion homeostasis for proper CNS operation, we aimed to explore the astrocytic homeostatic functions in ALS. For the purpose of our study, we generated a mouse with an ALS-like phenotype, SOD1 (G93A) mutation, and fluorescently labeled astrocytes, on the FVB/N background. Since the SOD1 model on the FVB/N background has not been thoroughly characterized yet and only recently became commercially available, we decided to examine astrocytes in both the brain and spinal cord in terms of their homeostatic functions and morphological changes, to fill the knowledge gap. In addition to elucidating the altered functional properties of astrocytes in ALS progression, our data can also serve as a stepping-stone for others working with the SOD1 model on FVB/N background.

## 2 Materials and methods

### 2.1 Animals

For all experiments, we used a transgenic mouse on FVB/N background with fluorescently labeled astrocytes, expressing human SOD1(G93A). As a control, we used their non-carrier littermates. To generate the mouse, we crossbred C57Bl/6J-Tg (SOD1*G93A)1Gur/J (JAX Strain: 004435) males with GFAP/EGFP (Nolte et al., 2001) females. The visualization of astrocytes in GFAP/EGFP mice is feasible due to the expression of enhanced green fluorescent protein (EGFP) under the control of the human glial fibrillary acidic protein (GFAP) promoter. To obtain the congenic strain, mice were backcrossed to FVB/N background for at least 10 generations. These mice are further termed as SOD1/GFAP/EGFP and CTRL/GFAP/EGFP. In behavioral testing, we also employed the transgenic mice overexpressing human SOD1(G93A) (JAX Strain: 004435 C57BL/6J-Tg (SOD1*G93A)1Gur/J) and their non-carrier littermates (Gurney et al., 1994). For these mice, we used the abbreviation SOD1/C57Bl6 and CTRL/C57Bl6, respectively.

All procedures involving the use of laboratory animals were performed following the European Communities Council Directive 24 November 1986 (86/609/EEC) and animal care guidelines approved by the Institute of Experimental Medicine, Academy of Sciences of the Czech Republic (Animal Care Committee in April 2019; approval number 40/2019). All efforts were made to minimize both the suffering and the number of mice used.

### 2.2 Behavioral testing

We conducted the wire grid hang test and the rota-rod test (Mouse RotaRod NG 47650, Ugo Basile, Italy) to assess muscle strength, function, and coordination throughout the disease. Weight was also measured as an additional parameter of the symptom progression. Testing consisted of a single three-attempt session every week, beginning at P30, and lasting for 14 weeks. Before the experiment, all mice were trained. Data are presented as mean or mean ± standard error of the mean (SEM) for n mice. Repeated measures of two-way ANOVA with Holm-Sidak’s multiple comparison correction were used to analyze the differences between groups.

#### 2.2.1 Wire grid hang test

Each mouse was placed on a custom-made wire lid, approximately 60 cm above a wood chip covered bottom, and turned upside down. The latency to fall was measured. At the beginning of the testing period, we trained each mouse three consecutive times for at least 180 s. In the experimental session, the mouse had three attempts to hold on to the lid. We noted the best score out of the three with a maximum of 180 s.

#### 2.2.2 Rotarod test

The mouse was placed on a stationary rod facing against the direction of rotation. The rod started rotating at a constant speed of 15 rpm, and the latency to fall was measured. Each mouse was trained three consecutive times of at least 180 s at 5, 10, and 15 rpm speed. In the experimental session, the mouse had three attempts to remain on the rod. We noted the best score out of the three with a maximum of 180 s.

### 2.3 Immunohistochemistry and image analysis

To obtain tissue for immunohistochemical analyses, the mice were deeply anesthetized with pentobarbital (PTB) (100 mg/kg, i.p.) and perfused transcardially with 20 mL of saline solution followed by 20 mL of cooled 4% paraformaldehyde (PFA) in 0.1 M phosphate buffer. The brains and spinal cords were then postfixed overnight with PFA and treated with a sucrose gradient (ranging from 10 to 30%) for cryoprotection. Coronal 30-μm-thick slices were prepared using a cryostat (Leica CM1850, Leica Microsystems, Wetzlar, Germany). For immunohistochemical staining, the slices were washed in a phosphate buffer saline followed by blocking of the non-specific binding sites with 5% Chemiblocker (Millipore, Billerica, MA), and 0.2% Triton in phosphate buffer saline. The blocking solution was also used as the diluent for the antisera. The slices were incubated with the primary antibodies overnight, and the secondary antibodies were applied for 2 h at 4–8°C. The following antibodies were used: anti-Kir4.1 (dilution 1:300; Alomone Labs, Jerusalem, IL; catalog number: APC-035-GP), goat anti-Guinea Pig IgG conjugated with Cy3 (dilution 1:200; Chemicon, Temecula, CA, United States; catalog number: AP108C). Cell nuclei were visualized by DAPI staining (Merck, Darmstadt, Germany). Following staining, the slices were mounted, and the fluorescence signal was imaged and scanned using a spinning disk microscope (Olympus, SpinSR10) equipped with dry UPLXAPO 40× objective.

The areas corresponding to cells with endogenous EGFP expression under GFAP promoter were quantified using FIJI image processing software (ImageJ 2.9.0/1.53t) (Schindelin et al., 2012). Segmentation was performed by thresholding the EGFP channel and the fluorescence-positive area was measured and related to the area of primary and secondary motor and primary somatosensory cortex or spinal ventral horn area. The area corresponding to EGFP reflects both the increased number of GFAP-expressing cells and the enlargement of astrocytic soma and the thickening of their processes. A similar thresholding approach for segmentation was also used for the Kir4.1 antibody signal. The immunopositive regions were segmented using the threshold dialog and the mean gray values were calculated in the segmented area. The statistical analysis of the differences among groups was performed using unpaired t-test. Error bars in plots represent SEM.

### 2.4 Preparation of acute brain and spinal cord slices for functional measurements

Experiments were performed on acute brain and spinal cord slices of SOD1/GFAP/EGFP and CTRL/GFAP/EGFP mice at the age of 4 months. The mice were deeply anesthetized with PTB (100 mg/kg, i.p.), and perfused transcardially with a cold (4–8°C) isolation buffer. Brains and spinal cords were dissected and placed into a cold isolation buffer (4–8°C), oxygenated with 95% O_2_ and 5% CO2 (Carbogen, Siad, Branany, Czech Republic). The spinal cord was then embedded into the low-melting agarose (Sigma-Aldrich). Brain and spinal cord coronal slices (300 μm for 3D-morphometry and 400 μm for the TMA method) were cut using an HM650 V vibratome (MICROM International GmbH, Waldorf, Germany). The slices were then incubated for 40 min at 34°C in the isolation solution. After the incubation period, the slices were kept at room temperature (23–25°C) in artificial cerebrospinal fluid (aCSF).

### 2.5 Experimental solutions for functional measurements

The compositions of all experimental solutions are listed in Table 1. All solutions were equilibrated with 95% O_2_ and 5% CO_2_ (Carbogen, Siad, Branany, Czech Republic) to a final pH of 7.4, while the osmolality was measured using a vapor pressure osmometer (Vapro 5,520, Wescor, Logan, United States).

**Table 1:** Contents of experimental solutions.

Compounds	aCSF [mM]	Isolation solution [mM]	20 mM aCSF_K+_[mM]	50 mM aCSF_K+_[mM]
NaCl	122	–	105	75
NMDG	–	110	–	–
KCl	3	2.5	20	50
NaHCO_3_	28	24.5	28	28
Na_2_HPO_4_	1.25	1.25	1.25	1.25
Glucose	10	20	10	10
CaCl_2_	1.5	0.5	1.5	1.5
MgCl_2_	1.3	7	1.3	1.3
Osmolality (mOsmol/kg)	~300	~300	~300	~300

NMDG, *N*-methyl-d-glucamine; aCSF, artificial cerebrospinal fluid; 20 or 50 mM aCSF_K+_, artificial cerebrospinal fluid with elevated K^+^ concentration.

### 2.6 Three-dimensional confocal morphometry

Time-dependent changes in astrocyte volume and morphology were studied using 3D-confocal morphometry in the motor cortex and in the ventral horns of the spinal cord. The method was previously described in Awadova et al. (2018) and Pivonkova et al. (2018). Briefly, brain or spinal cord slices were carefully placed into the recording chamber and mounted on the stage of a confocal microscope. The slices were held down with a U-shaped platinum wire with a grid of nylon threads. The recording chamber was continuously perfused with recording solutions (aCSF or aCSF_K_^+^) via peristaltic pump PCD 31.2 (Kouril, Kyjov, Czech Republic) at a rate of ~7.5 mL/min. The exchange of solutions in the recording chamber took place within 2 min. All measurements were performed at room temperature. Fluorescence images were acquired on a multiphoton laser scanning microscope FV1200MPE (Olympus) with 60× LUMPLFLN water objective (NA = 1.0, WD 2 mm). EGFP fluorescence was excited in a 2-photon absorption mode at 950 nm by a tunable Ti-Sapphire laser system MaiTai DeepSee (Spectra Physics, CA). A fluorescence signal selected by 495–540-nm band-pass emission filter was detected by GaAsP detector. Cortical astrocytes (layers II – V of the motor cortex) and astrocytes in the anterior horns of the spinal cord were imaged in a z-stack with a uniform spacing of 0.5 μm and a resolution of 512 × 512 pixels with the voxel size 0.41 × 0.41 × 0.5 μm.

The signal acquisition was set so that the cell body did not contain oversaturated pixels. The imaging was done as follows: three full 3D stacks of images were acquired in the isotonic aCSF solution to assess the rate of image acquisition-induced photobleaching by linear correction. During the cell volume changes induced by aCSF_K+_ 20 mM or 50 mM treatment, individual 3D images were acquired in 5-min intervals, 4 times in total. Finally, the cell volume recovery was checked following the re-application of isotonic aCSF solution (washout) after 20 and 40 min.

Measurements of fluorescence intensity (FI) and soma size (Ss) were performed in Fiji image processing software (ImageJ 2.9.0/1.53t)^1^ (Schindelin et al., 2012). Variations of FI due to cell volume changes were monitored as follows: *x–y–z* image stacks acquired in each individual time point were reduced to 2D frames by an average intensity projection along the *z*-axis. These reduced images were composed to a time stack and aligned with StackReg (ImageJ plugin by Philippe Thévenaz, Biomedical Imaging Group, Swiss Federal Institute of Technology, Lausanne). Integral fluorescence intensities within a circular ROI of ∼2 μm in diameter located within the cell soma were measured. Average intensity projections of the recorded 3D stacks were segmented by an automatic threshold based on the Isodata Algorithm (Ridler and Calvard, 1978). The cell area in the frame containing the segmented cell body was treated as the 2D measure of the Ss. Soma volume (*Vs*) is then proportional to √Ss3, assuming isotropic volume changes. For each CNS part (brain or spinal cord) at least 3 mice were used, with a minimum of 2 slices per mouse. Changes in the total astrocyte volume are presented as the mean ± SEM. Statistical significance was determined using two-way ANOVA. Differences between the groups were considered statistically significant when *p* < 0.05 (^*^), very significant when *p* < 0.01 (^**^), and extremely significant when *p* < 0.001 (^***^).

### 2.7 TMA method

The slices were placed into the experimental chamber connected with upright Zeiss Axioscope microscope (Zeiss, Germany) and manipulators (Luigs and Neumann, Germany). Measurements were performed at room temperature (22–24°C) at a depth of 200 μm, during a continuous perfusion with aCSF solution enriched with 0.1 mM tetramethylammonium ion (TMA^+^) and saturated with carbogen. Recordings were carried out every 5 min.

The ECS diffusion parameters, volume fraction *α* (*α* = ECS volume/total tissue volume), tortuosity *λ* (*λ*^2^ = free diffusion coefficient/apparent diffusion coefficient) and non-specific uptake (k’ [s^−1^]), were measured in acute brain and spinal cord slices using the real-time iontophoretic method (RTI) as described in detail in Nicholson and Phillips (1981) and Sykova and Nicholson (2008). In brief, the extracellular marker TMA^+^ (molecular weight 74.1 Da) was introduced into the tissue through an iontophoretic micropipette. The TMA^+^ imitated the diffusion of small ions and molecules in the ECS, and its time-dependent changes in the extracellular concentration were measured by double-barreled ion-selective microelectrodes (ISM). The manufacturing of double-barreled TMA^+^-ISMs was described in detail previously (Syková, 1992). Briefly, the reference barrel was filled with 150 mM NaCl and an ion-sensitive barrel contained an ion exchanger IE190 (WPI, Inc., Sarasota, United States, RRID: SCR_008593) at the tip, and 100 mM TMA^+^ solution as a backfilling solution. Prior to each experiment, the TMA^+^-ISMs were calibrated in a series of five different solutions with increasing TMA^+^ concentrations: 0.1, 0.3, 1.0, 3.0, and 10.0 in a background of 3 mM KCl and 150 mM NaCl. The TMA^+^ signals were fitted to the Nikolski equation to acquire the slope and interference of each ISM (Nicholson, 1993). The iontophoretic micropipettes backfilled with 100 mM TMA chloride were glued to individual ISMs with a tip separation of 50–100 μm. Prior to the experimental measurements, the ISMs arrays were calibrated in 0.3% agar gel (Merck, Germany). The 20 nA bias current was continuously applied (Single Channel Iontophoresis Generator ION-100; Dagan Corporation, Minneapolis, Minnesota, United States) to maintain a constant electrode transport number. A current step of 200 nA and 24 s duration (stimulator master 8, A.M.P.I, Jerusalem, Israel) generated a diffusion curve. The obtained diffusion curves were analyzed by a non-linear curve fitting simplex algorithm, operating on a modified diffusion equation using the VOLTORO program (kindly provided by C. Nicholson, New York University School of Medicine, USA, unpublished data) to acquire the values of the electrode transport number (*n*) and the free diffusion coefficient of TMA^+^ (*D*). Knowing the *n* and *D* values, the parameters *α*, *λ* and k’ could be determined from the measured tissue. The measurements were performed at room temperature (22–24°C) in aCSF, and the obtained values were averaged. Diffusion measurements were performed in the brain cortex and the anterior horn of the lumbar part of the spinal cord at a depth of 200 μm. The stable control values were acquired in the aCSF, followed by a 20 min application of increased potassium concentrations (20 mM or 50 mM aCSF_K+_) and by a 40 min wash-out in aCSF. The diffusion curves were captured and analyzed every 5 min.

Data are expressed as mean ± SEM, where *n* represents the number of slices/measurements. Statistical analyses were performed using Graph Pad statistical package^2^ – between experimental groups using two-way ANOVA analysis, followed by Sidak’s multiple comparison test and one-way ANOVA with Dunnet’s multiple comparison test to analyze changes within a single group (CTRL and SOD1 separately). Differences between the groups were considered statistically significant when *p* < 0.05 (^*^), very significant when *p* < 0.01 (^**^), and extremely significant when *p* < 0.001 (^***^).

### 2.8 Elemental analysis of CSF

#### 2.8.1 CSF isolation

The experiment was performed using SOD1/GFAP/EGFP and CTRL/GFAP/EGFP mice at the age of 4 months. To isolate the cerebrospinal fluid, we used modified protocol from (Kaur et al., 2023). Briefly, we used borosilicate glass capillaries (Sutter Instruments, Novato, CA, United States) and P-97 Brown-Flaming puller (Sutter Instruments, Novato, CA, United States) and prepared the capillary, using pulling settings as specified in the protocol. The mice were initially anesthetized with 3% isoflurane (Abbot, IL, United States) and maintained at 1–2% isoflurane using a vaporizer (Tec-3, Cyprane Ltd., Keighley, United Kingdom). We omitted the use of a stereotactic instrument but followed the protocol with cisterna magna exposure and then the CSF extraction. We looked for any traces of blood contamination and used only clear samples for further analysis. The average amount of isolated CSF per mouse was 15 μL. The body temperature of the mouse was maintained at 37 ± 1°C using a heating pad throughout the procedure and the mouse was sacrificed immediately after.

#### 2.8.2 Elemental analysis

The ion concentration in the cerebrospinal fluid was determined using inductively coupled plasma optical emission spectroscopy (ICP-OES) coupled with an electrothermal evaporation (ETV) unit. ICP-OES is a widely used highly sensitive analytical method capable of determining most elements. In brief, a sample in the form of a solution is converted to a fine aerosol, which is then carried by a stream of argon into a high-temperature argon plasma, where evaporation, atomization, and excitation take place. The characteristic radiation emitted by the excited atoms is then processed in the spectrometer. The ETV is an alternative sample introduction system, which allows direct analysis of solid or liquid samples. The sample was weighed in a graphite boat and heated in a graphite furnace using an optimized temperature program in an argon atmosphere, with the maximum achievable temperature of 3,000°C. The addition of a small amount of CCl_2_F_2_ into the furnace facilitates the conversion of analytes to more volatile forms, thus ensuring complete analyte evaporation. The generated dry aerosol is carried by argon stream directly into the plasma. This approach offers lower detection limits and matrix-independent calibration, thus allowing the analysis of smaller sample amounts, thanks to nearly loss-free transport of sample directly into the plasma. For analysis, 10 μL of cerebrospinal fluid was pipetted into a graphite boat and the sample amount was checked by weighing it. Due to low sample volumes, only one replica per sample was possible. An Arcos I (Spectro, Kleve, Germany) ICP-OES instrument coupled to an ETV 4000c unit (Spectral Systems, Fürstenfeldbruck, Germany) was used for the analysis.

## 3 Results

### 3.1 Behavioral testing revealed phenotypical differences between C57Bl/6 and FVB/N mSOD1 mice

**Figure 1:**
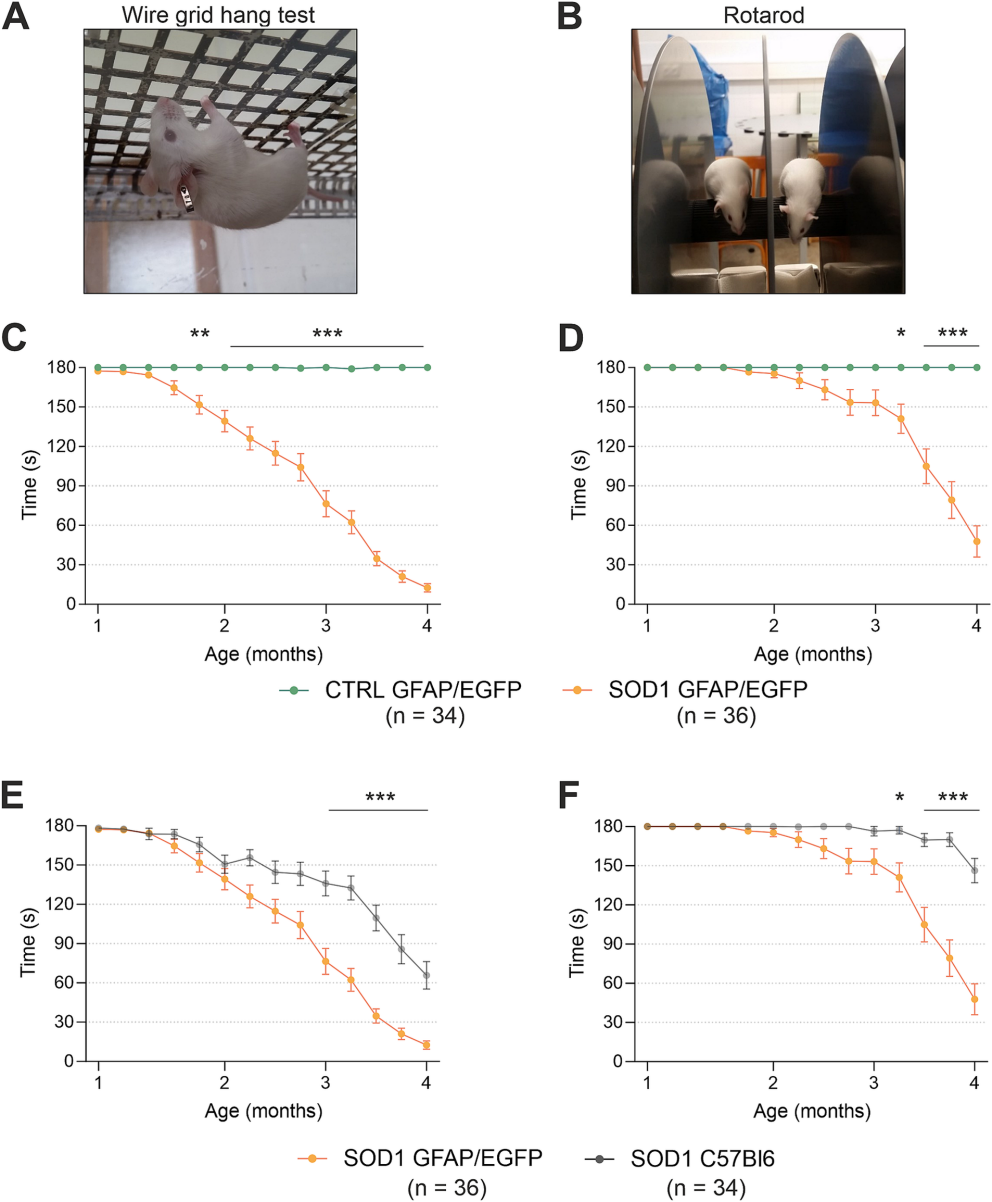
Evaluation of pathology by behavioral testing. **(A)** Representative picture of the wire grind hang test execution. **(B)** Representative picture of the Rotarod test execution. **(C)** The hanging wire test confirmed the motor strength decline in SOD1/GFAP/EGFP mice compared to CTRLs. **(D)** Rotarod measurements analysis revealed the decline of motor coordination in SOD1/GFAP/EGFP mice compared to CTRLs and thus confirmed the phenotype. **(E)** Comparison of motor strength revealed significantly faster progression in SOD1 GFAP/EGFP mice compared to the initial SOD1/C57Bl6. **(F)** Similar results were observed in Rotarod results, showing a faster decrease of motor coordination in SOD1 GFAP/EGFP mice. Data are presented as mean ± SEM. n = the number of mice. Repeated measures of two-way ANOVA with Holm–Sidak’s multiple comparison correction were used to analyze the differences between groups. Differences between the groups were considered statistically significant when *p* < 0.05 (*), very significant when *p* < 0.01 (**), and extremely significant when *p* < 0.001 (***).

To confirm the phenotype of our animal model created by crossbreeding of C57Bl6 SOD1(G93A) and GFAP/EGFP mice (Nolte et al., 2001), we conducted the hanging wire motor test (Figure 1A) and Rotarod test (Figure 1B). We tested a comparable number of mice for all four groups: SOD1/C57Bl6; CTRL/C57Bl6; SOD1/GFAP/EGFP and CTRL/GFAP/EGFP with an equal distribution of males and females within each group.

First, we compared SOD1/GFAP/EGFP (*n* = 34) and CTRL/GFAP/EGFP (*n* = 36) mice (Figures 1C,D). Based on the results, we can confirm that our crossbred mice manifest typical ALS symptoms such as loss of strength in both forelimbs and hind limbs and poor motor coordination (Gurney et al., 1994). Mutants compared to controls performed significantly worse in both tests from 2 months of age, which is generally considered an onset point for the SOD1(G93A) model (Mead et al., 2011; Gerber et al., 2012; Mancuso et al., 2011). The hanging wire test showed continuous strength decline, with the mice being almost unable to hold themselves on the grid at the end stage, which suggests severe strength loss, mainly in the hind limbs (Figure 1C). The Rotarod measurements showed significantly impaired motor coordination in mutant mice starting around 3 months of age (Figure 1D). Our results are in agreement with the data from SOD1(G93A) mice from the standpoint of the onset. Nevertheless, we decided to compare the overall performance of the two strains as well, to see whether the different backgrounds have any effect on the phenotype.

Interestingly, we discovered significant differences between the SOD1/GFAP/EGFP and SOD1/C57Bl6 mice performance during the symptomatic stage (Figures 1E,F). The strength in both forelimbs and hind limbs was comparable until the onset, but then the gross phenotype of SOD1/GFAP/EGFP mice worsened much faster and their performance at 3 months of age was comparable to that observed in the final stage (4 months of age) of SOD1/C57Bl6 and continued to decline (Figure 1E). The motor coordination tested by Rotarod also seemed to be more impaired in SOD1/GFAP/EGFP mice. Again, their performance at 3 months of age was comparable to the final stage (4 months of age) of SOD1/C57Bl6 (Figure 1F).

Overall, the onset point remains similar for both strains; however, the course of the disease seems to differ between the SOD1/GFAP/EGFP and SOD1/C57Bl6 mice. The SOD1/GFAP/EGFP mice decline faster and they reach the “final stage” values of the original model about a month earlier. Our data thus suggest that a different genetic background does not affect the onset of the disease manifestation but rather affects the rate of disease progression or worsening of the overall symptoms.

### 3.2 Fluorescence analysis confirmed astrogliosis

**Figure 2:**
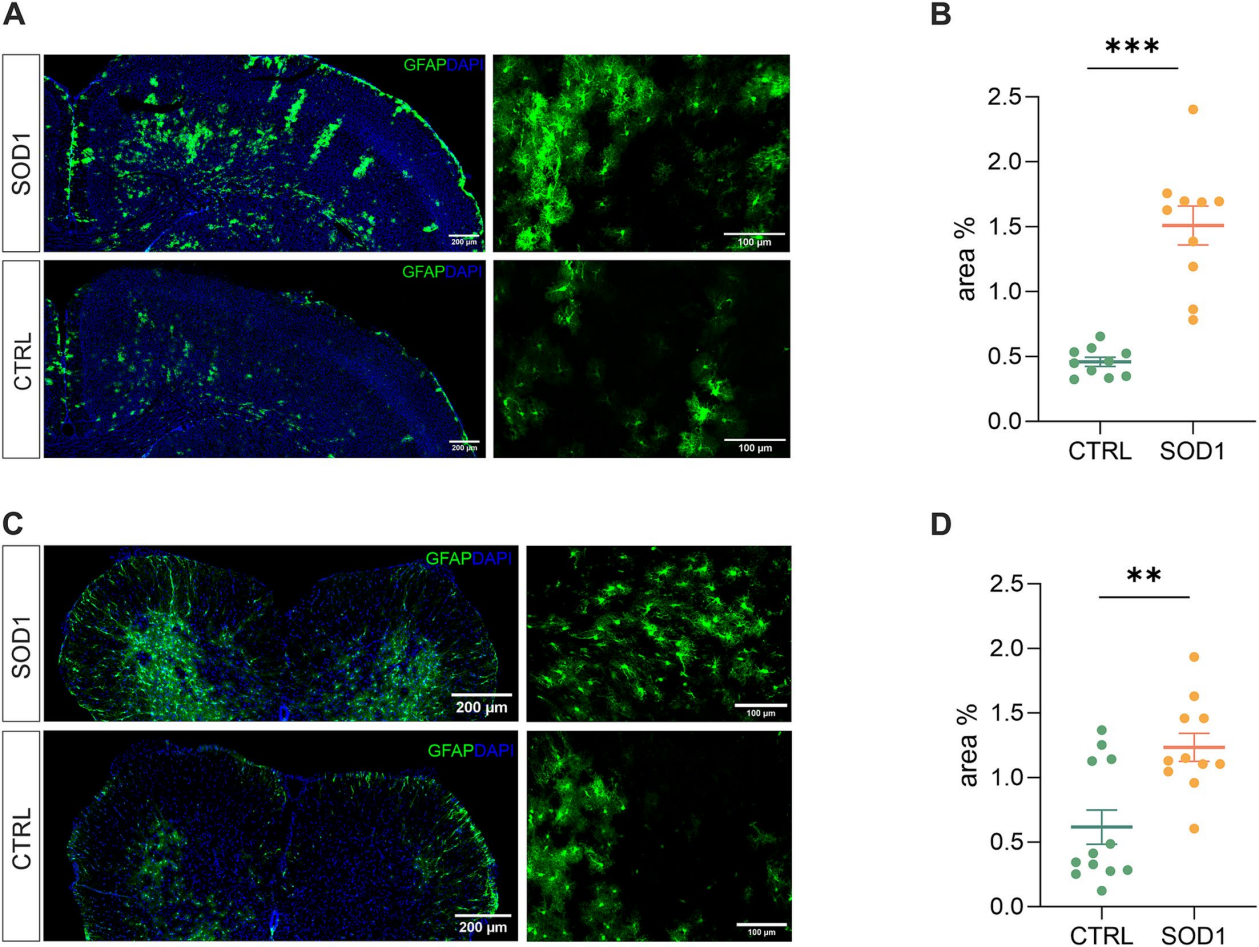
Assessment of astrocyte reactivity based on GFAP expression levels. **(A)** Representative tile scans of motor cortex used for fluorescence analysis. The SOD1 cortex is showing signs of astrogliosis. **(B)** Fluorescence analysis of astrogliosis in the motor and somatosensory cortex of SOD1 and CTRL mice showed astrogliosis in SOD1 mice. **(C)** Representative pictures of astrocytes in the ventral horns of the spinal cord. **(D)** Fluorescence analysis of astrogliosis in the spinal cord confirmed a gliosis and morphological shift of astrocytes toward the reactive shape. Data are presented as mean ± SEM. n = the number of brain hemispheres or spinal cord slices, respectively. Unpaired *t*-test was used to analyze the differences between groups. CTRL, control mice on the FVB/N background; SOD1, superoxide dismutase transgenic mice on the FVB/N background. Differences between the groups were considered statistically significant when *p* < 0.05 (*), very significant when *p* < 0.01 (**), and extremely significant when *p* < 0.001 (***).

To define the state of astrocytes in the SOD1/GFAP/EGFP model, we took advantage of their eGFP-labeling and performed a fluorescence analysis looking for the signs of astrogliosis in both the brain and spinal cord. Astrogliosis is marked by cellular hypertrophy, shortened cell processes and increased GFAP expression, and was described in the spinal cord of the original SOD1(G93A) model on the C57BL6 background (Miller et al., 2017; Guttenplan et al., 2020). The regions of interest for fluorescence analysis were the motor-the somatosensory cortex, and the ventral horn of the lumbar spinal cord. To ensure we analyzed the whole area of interest, we took advantage of the tile scanning technique (see Figures 2A,C). 120 ± 3 days old mice were used for the analysis.

Our results in the motor and somatosensory cortex (Figure 2B) revealed a significantly larger fluorescent area in the SOD1 mice, and we observed the same situation in the ventral horns of the lumbar spinal cord (Figure 2D). This suggests a higher GFAP positivity (thus a visible change in morphology and/or increase of GFAP-positive cells) caused by ALS-like pathology. These results confirm that astrocytes become activated in both the brain and spinal cord during the disease progression in the model on the FVB/N background. This is consistent with the results from the SOD1/C57Bl6 original model, in which astrocytic activation is considered one of the disease hallmarks.

### 3.3 Spinal SOD1/GFAP/EGFP astrocytes exhibit disrupted volume regulation during higher potassium concentrations

**Figure 3:**
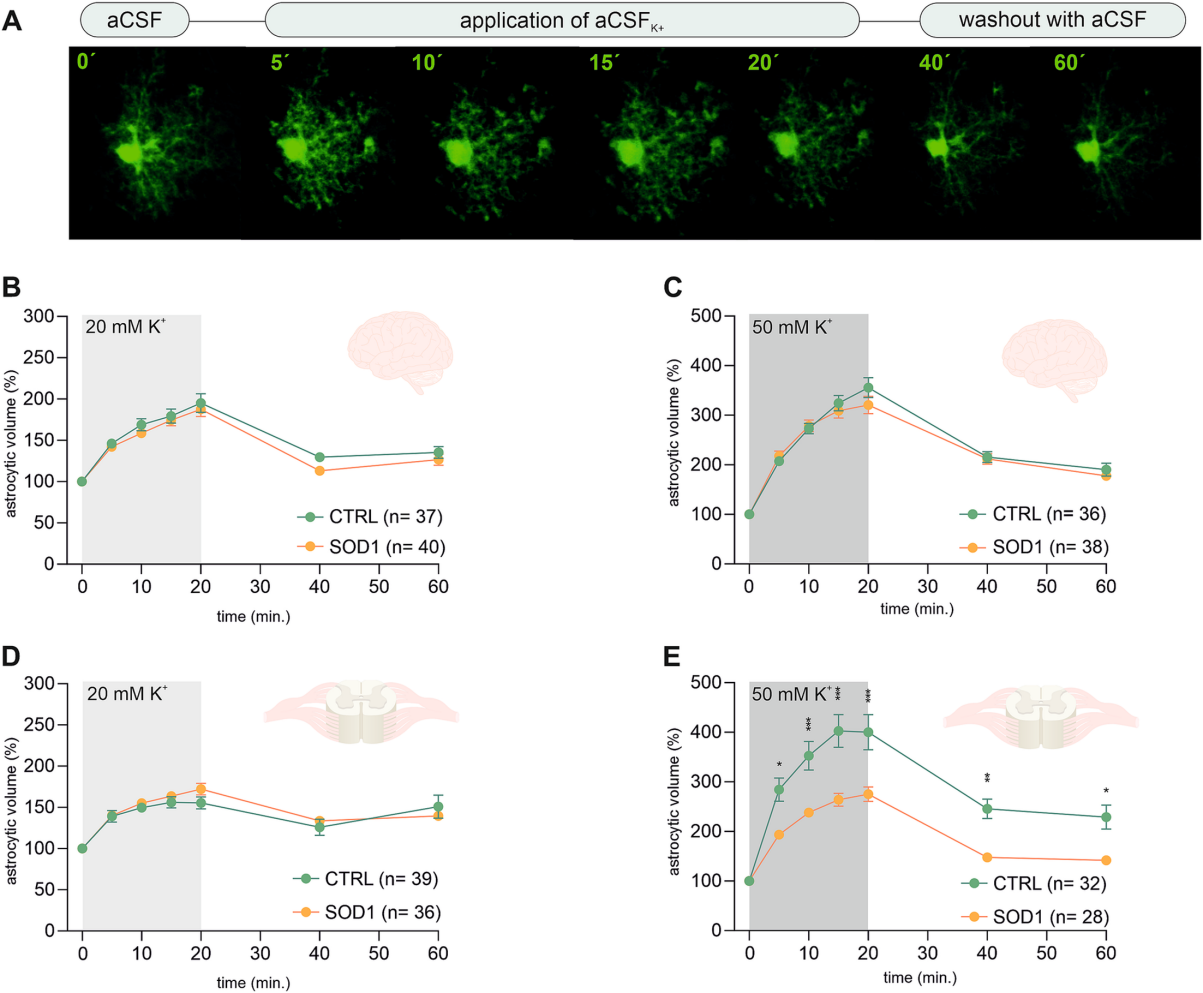
Swelling of astrocytes in response to different potassium concentrations. **(A)** Superimposed confocal images of an EGFP-labeled cortical astrocyte were recorded throughout the measurement. A control image was taken during the application of artificial cerebrospinal solution (aCSF). The volume of astrocytes was recorded every 5 min during the application of aCSF_K+_ for 20 min. The last 2 stacks of astrocyte images were recorded at an interval of 20 min during 40-min in aCSF. **(B,C)** The time course of volume changes of cortical **(B)** and spinal **(C)** astrocytes throughout the measurement in 20 mM aCSF_K+_. **(D,E)** Time course of volume changes of cortical **(D)** and spinal **(E)** astrocytes throughout the measurement in 50 mM aCSF_K+_. Data are presented as mean ± SEM. n = number of cells. Two-way ANOVA with Holm–Sidak’s multiple comparison correction were used to analyze the differences between groups CTRL, control mice on the FVB/N background; SOD1, superoxide dismutase transgenic mice on the FVB/N background.

The analysis of EGFP fluorescence revealed morphological changes in both cortical and spinal astrocytes suggesting their shift toward an activated state. We were interested to see whether this shift has affected astrocytic ability to swell, which is connected to potassium uptake that contributes to maintaining potassium homeostasis. Potassium levels tend to be higher during pathological stages, so we conducted an experiment measuring astrocytic volume during exposure to different potassium concentrations. We used 20 mM and 50 mM aCSF_K+_ solutions to evoke elevated potassium levels occurring, e.g., during closed brain injury and ischemia, respectively (Rossi et al., 2007; Muller and Somjen, 2000; Pietrobon and Moskowitz, 2014). The measurements were conducted using acute brain and spinal cord slices from SOD1/GFAP/EGFP and CTRL/GFAP/EGFP mice exposed to either 20 mM or 50 mM aCSF_K+_ for 20 min followed by a 40-min application of aCSF (further termed as washout). Changes in astrocytic volume were recorded every 5 min during the application and every 20 min during the washout (see Figure 3A). Astrocyte volume at *t* = 0 was set to 100% and the swelling was expressed relative to this baseline as an increase in percentage. 120 ± 3 days old mice were used for the analysis.

Volume changes of cortical astrocytes were measured specifically in the motor and the somatosensory cortex of acute brain slices. Surprisingly, the measurements and following data analysis revealed that SOD1 cortical astrocytes can handle higher potassium levels with the same efficacy as healthy astrocytes during both 20 and 50 mM K^+^ application (Figures 3B,C) despite the observed astrogliosis. After initial measurements cells swelled up to ~190% and almost ~340%, respectively. All cells were able to recover their volume in the following 40 min, suggesting there is no impairment in potassium level maintenance.

Spinal astrocytes were measured specifically in the ventral horns of the spinal cord. The results of our measurements show that the SOD1 spinal astrocytes have unimpaired swelling capacity in the 20 mM K^+^ environment (Figure 3D) as they swell comparable to healthy cells. The higher potassium concentration; however, represents a challenge. The SOD1 astrocytes swelled significantly less in the 50 mM K^+^ than the CTRL astrocytes throughout the whole application (Figure 3E). While the SOD1 astrocytes swelled up to only ~275%, the CTRL astrocytes swelled to ~400% of their original volume. The difference in swelling in the high potassium environment suggests inefficient uptake of the extracellular potassium, which could result in potassium homeostasis imbalance in the SOD1 spinal cord. Despite the extensive swelling, both SOD1 and CTRL astrocytes were able to recover their volume during the washout, even though in the CTRL cells this was only partially.

### 3.4 A higher potassium concentration alters the extracellular space diffusion parameters in the SOD1/GFAP/EGFP spinal cord

To investigate alterations in the dynamics of the ECS diffusion properties, we used the RTI method and measured the brain and spinal cord of 120 ± 3 day old mice. We hypothesized that the alterations detected in SOD1/GFAP/EGFP mice astrocyte morphology and their functional properties analyzed by 3D-morphometry could be manifested as variations in the ECS parameter values. To address this, we employed the same experimental protocol as outlined earlier. Briefly, brain and spinal cord slices from SOD1/GFAP/EGFP and CTRL/GFAP/EGFP were exposed to two distinct aCSF_K+_ concentrations (20 and 50 mM) separately, for 20 min, followed by a 40-min washout period with aCSF. The alterations in ECS parameters from the initial values determined before the application were monitored at 5-min intervals throughout both the application phase (20 min) and the subsequent washout period (40 min). The quantification of the ECS parameters volume fraction (*α*) and tortuosity (*λ*) was done in the cerebral milieu of the motor cortex and the ventral horns of the spinal cord.

**Figure 4:**
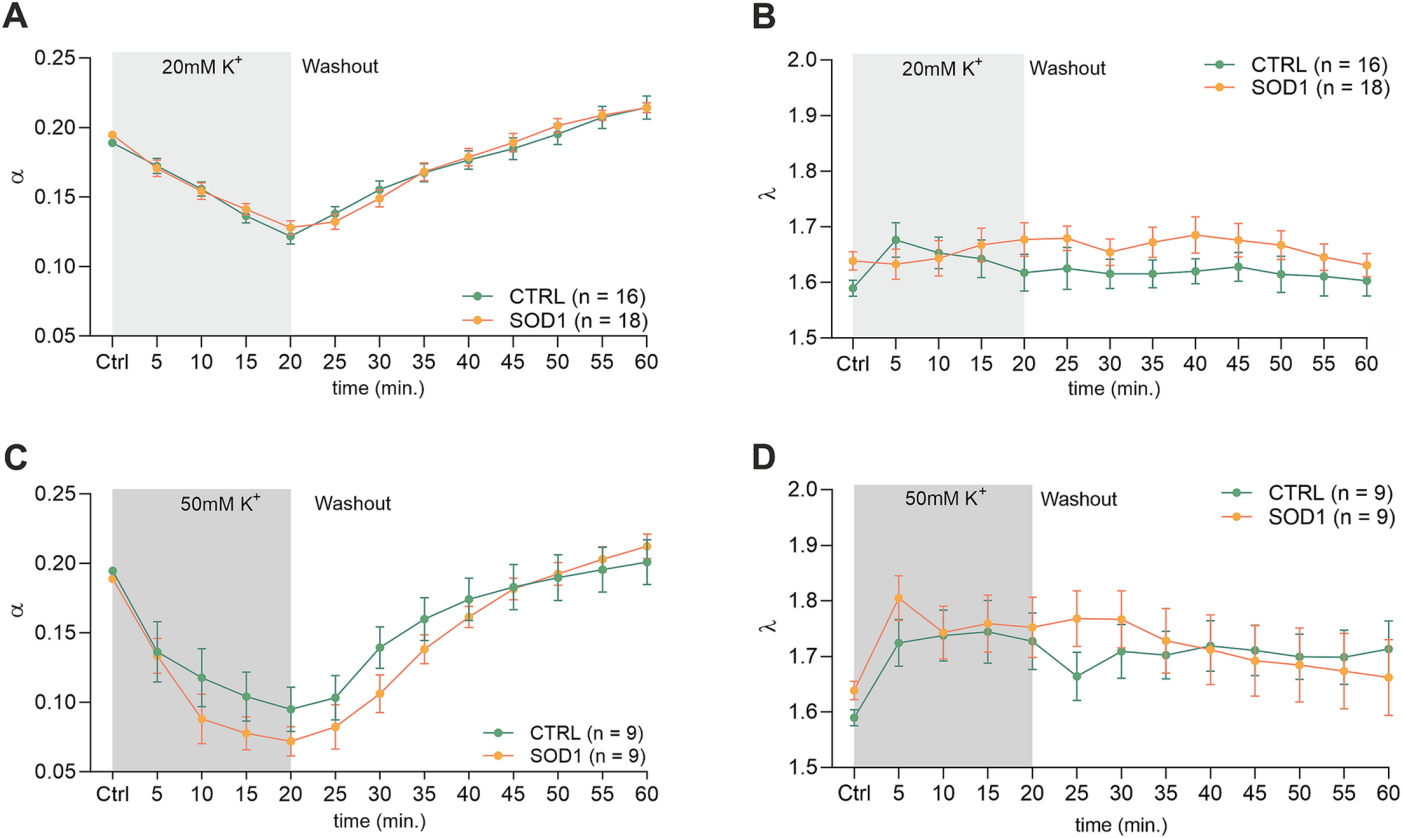
Measurements of ECS parameters in mouse acute brain slices. Averaged data of *α* – volume fraction and *λ* – tortuosity measured in CTRL and SOD1 mice at resting conditions (22–24°C, Ctrl) and at 5-min intervals during application of 20 mM **(A,B)** or 50 mM **(C,D)** aCSF_K+_ and washout (aCSF). Data are presented as mean ± SEM. n = the number of slices. Two-way ANOVA with Holm–Sidak’s multiple comparison correction were used to analyze the differences between groups Ctrl, control (initial measurements); CTRL, control mice on the FVB/N background; SOD1, superoxide dismutase transgenic mice on the FVB/N background.

The initial values in the cortex of CTRL mice (*α* = 0.189 ± 0.006, *λ* = 1.59 ± 0.05, *n* = 16, expressed as mean ± SEM) did not significantly differ from those acquired in SOD1 mice (*α* = 0.195 ± 0.006, *λ* = 1.64 ± 0.04, *n* = 18). Application of 20 mM aCSF_K+_ (Figures 4A,B) for 20 min induced cell swelling in both experimental groups, leading to a shrinkage of the ECS volume, manifested as a compensatory decrease of the ECS volume fraction (*α*: CTRL 0.122 ± 0.006, SOD1 0.128 ± 0.005, *p* < 0.0001), while no significant changes in tortuosity were detected (*λ*: CTRL 1.605 ± 0.03, SOD1 1.677 ± 0.03). During the 40 min washout, the ECS volume fraction fully recovered to initial values in both CTRL and SOD1 mice or even exceeded them, however, no significant differences between groups were found.

The exposure of brain slices to 50 mM aCSF_K+_ (Figures 4C,D) revealed a statistically significant decrease in the volume fraction in both groups (*p* < 0.001) compared to the initial values, with no significant difference observed between CTRL (*α* = 0.072 ± 0.01, *n* = 9) and SOD1 mice (*α* = 0.095 ± 0.02, *n* = 9). Tortuosity was not significantly affected by the 50 mM aCSF_K+_ application, either in CTRL or SOD1 mice; the maximum values reached in the 20th min of application did not differ between CTRL and SOD1 mice either (*λ*: 1.73 ± 0.05 and 1.75 ± 0.05, respectively). The subsequent washout procedure demonstrated a complete recovery of the volume fraction toward the end of the experiment in both groups.

**Figure 5:**
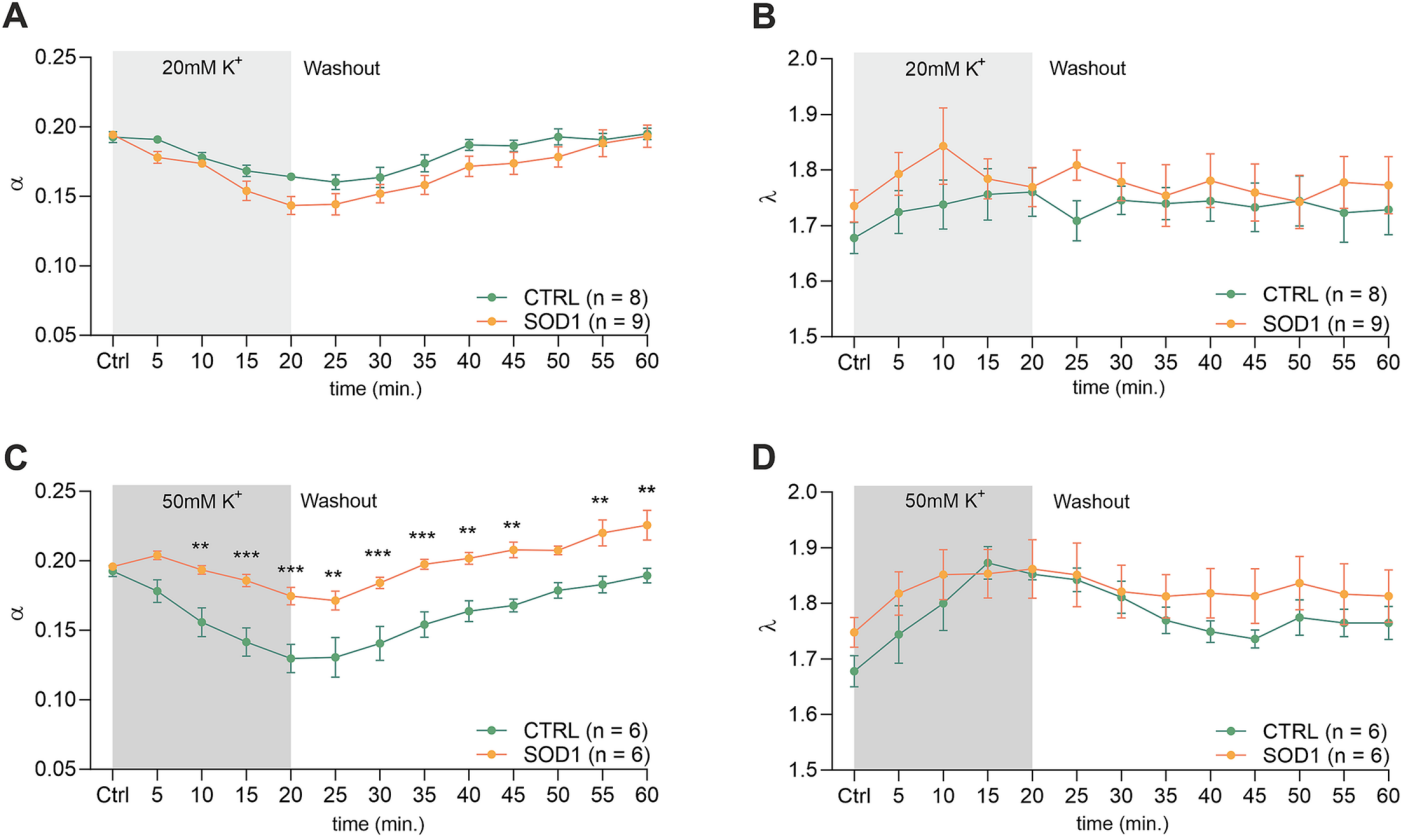
Measurements of ECS in acute spinal cord slices. Averaged data of α – volume fraction and λ – tortuosity measured in CTRL and SOD1 mice at resting conditions (22–24°C, Ctrl), and at 5-min intervals during application of 20 mM **(A,B)** and 50 mM **(C,D)** aCSF_K+_ and washout (aCSF). Data are presented as mean ± SEM. n = the number of slices. Two-way ANOVA with Holm–Sidak’s multiple comparison correction were used to analyze the differences between groups Ctrl, control (initial measurements); CTRL, control mice on the FVB/N background; SOD1, superoxide dismutase transgenic mice on the FVB/N background. Differences between the groups were considered statistically significant when *p* < 0.05 (*), very significant when *p* < 0.01 (**), and extremely significant when *p* < 0.001 (***).

The analysis of values obtained prior to the application in the spinal cord showed that there were no differences in the initial values of the ECS volume fraction or tortuosity between the groups of mice (CTRL: *α* = 0.193 ± 0.007, *λ* = 1.68 ± 0.05; SOD1: *α* = 0.196 ± 0.003, *λ* = 1.75 ± 0.07). Following exposure of the spinal cord tissue to 20 mM aCSF_K+_, the volume fraction decreased from the initial values in both experimental groups (CTRL: *α* = 0.164 ± 0.003, *n* = 8, SOD1: *α* = 0.151 ± 0.008; *p* < 0.001, *n* = 9); the maximum decrease reached in the 20th min did not differ between the groups (Figures 5A,B). No changes from the initial values were detected in tortuosity (CTRL: *λ* = 1.76 ± 0.04; SOD1: *λ* = 1.81 ± 0.02). The washout resulted in full recovery of the ECS volume in both groups: CTRL (*α* = 0.195 ± 0.004) and SOD1 (*α* = 0.196 ± 0.009) after 40 min of aCSF application.

Exposure of the spinal cord slices to 50 mM aCSF_K+_ resulted in a more profound decrease in the ECS volume fraction in CTRL (*n* = 6) compared to SOD1 (*n* = 6) mice (*α* = 0.130 ± 0.01 and 0.171 ± 0.008, respectively). Moreover, this decrease was faster in CTRL mice (*p* < 0.0001 already in 10th min) than in SOD1 group (*p* = 0.03 in 20th min) (Figure 5C). Full recovery of ECS volume fraction was detected in CTRL mice (*α* = 0.189 ± 0.005), while the values at the end of the washout in SOD1 mice exceeded the initial values, indicating that the ECS volume after washout was larger than before application (*α* = 0.226 ± 0.011, *p* = 0.0006). To further analyze the inter-group differences in the ECS volume changes during the washout period, the *α* values during washout were expressed as a percentage of volume increase/decrease in relation to the value of *α* reached in the 20th min of application, which was set as 100%. At the end of the washout (40th min), the ECS volume reached 123 ± 10% of its initial value in CTRL mice and 259 ± 82% in SOD1 mice. However, this analysis did not show any significant differences between CTRL and SOD1 mice, most likely due to the huge variability in the recovery rate in individual experiments. Interestingly, perfusion with 50 mM aCSF_K+_ evoked a steep increase in tortuosity values in CTRL mice (*p* < 0.0005), while tortuosity in SOD1 mice remained unchanged (Figure 5D).

### 3.5 Downregulation of potassium channel Kir4.1 was confirmed by immunohistochemical analysis

**Figure 6:**
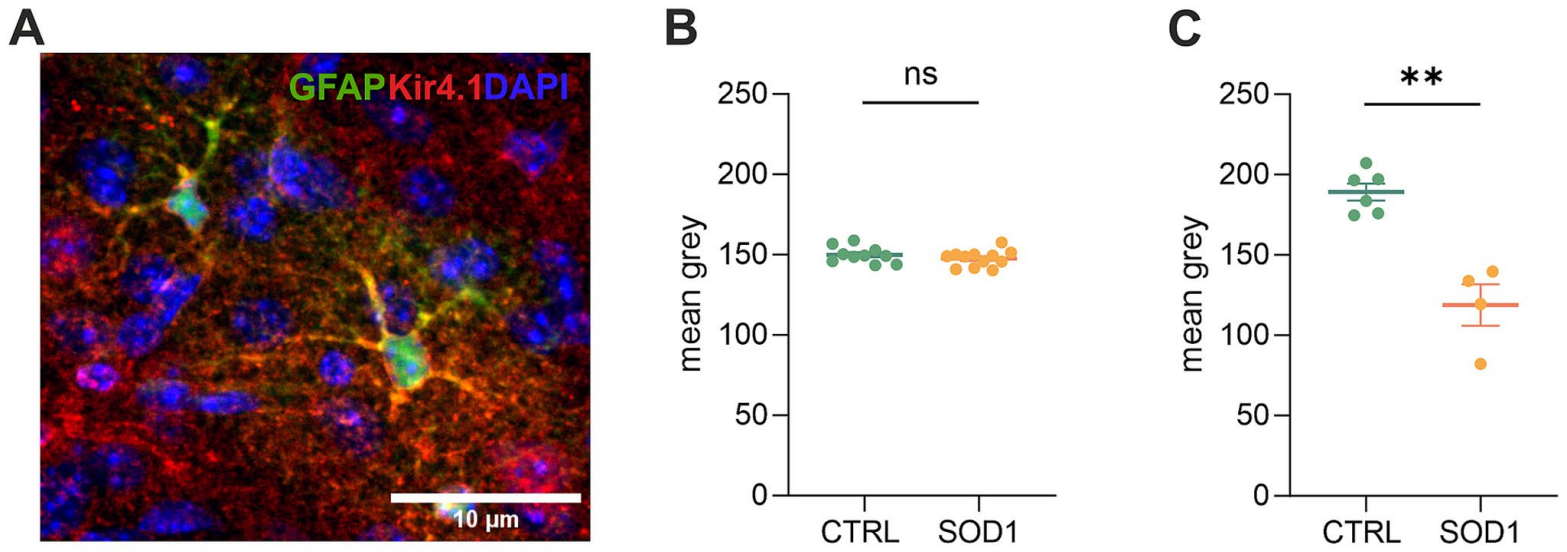
Immunohistochemical analysis of Kir4.1 expression. **(A)** A representative picture of Kir4.1 staining in the motor cortex. **(B)** Fluorescence analysis of Kir4.1 in the primary and secondary motor cortex and somatosensory cortex. **(C)** Fluorescence analysis of Kir4.1 in the ventral horns of the spinal cord. Data are presented as mean ± SEM. n = the number of brain hemispheres or spinal cord slices, respectively. Unpaired *t*-test was used to analyze the differences between groups. CTRL, control mice on the FVB/N background; SOD1, superoxide dismutase transgenic mice on the FVB/N background. Differences between the groups were considered statistically significant when *p* < 0.05 (*), very significant when *p* < 0.01 (**), and extremely significant when *p* < 0.001 (***).

Due to the observed differences in astrocyte swelling during higher potassium concentrations, we were interested whether this could be associated with the expression of Kir4.1 (Figure 6A), as these channels undergo downregulation in numerous CNS pathologies (Wilcock et al., 2009; Harada et al., 2013; Hanani and Spray, 2020). Kir4.1, predominantly expressed in astrocytes, is a subtype of Kir channels, which are crucial for maintaining the resting membrane potential and regulating K^+^ homeostasis in cells. To assess the expression, we employed immunohistochemistry followed by fluorescence analysis. We looked at the protein expression in the motor and the somatosensory cortex as well as in the spinal ventral horns of 120 ± 3 day old SOD1/GFAP/EGFP and CTRL/GFAP/EGFP mice. Our results suggest that there is no significant difference between Kir4.1 expression in the cortex, as the values we obtained were almost equal in both SOD1 and CTRL mice (Figure 6B). However, the fluorescence analysis in the ventral horns of the spinal cord showed significant downregulation of the Kir4.1 channel (Figure 6C). This downregulation could be associated with the lower swelling observed in spinal SOD1 astrocytes during the 50 mM aCSF_K+_ application.

### 3.6 Elemental analysis revealed low magnesium concentration in the cerebrospinal fluid of mutated mice

**Figure 7:**
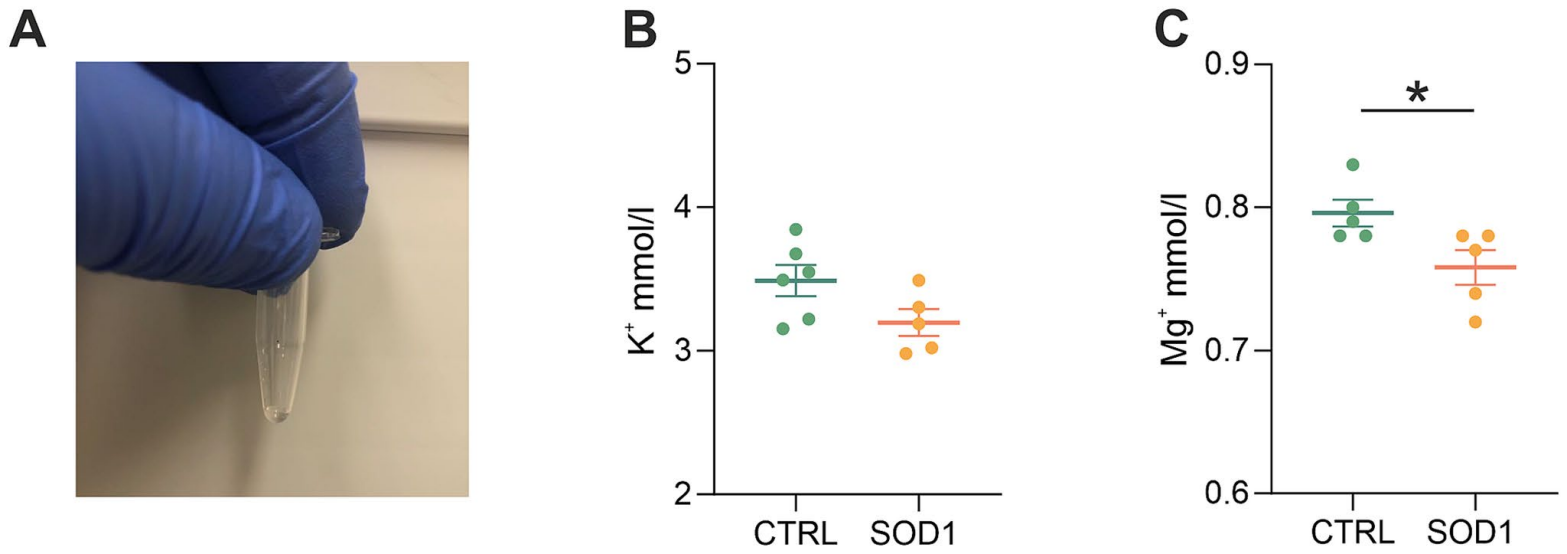
Elemental analysis of CSF isolated from GFAP/EGFP mice. **(A)** A sample of CSF demonstrating the purity of all samples used for the analysis. **(B)** A comparison of potassium concentrations in the CSF of control vs. SOD1 mice. **(C)** A comparison of magnesium levels in the CSF of control vs. SOD1 mice. Data are presented as mean ± SEM. n = the number of mice. Unpaired *t*-test with Welch’s correction was used to analyze the differences between groups. CTRL, control mice on the FVB/N background; SOD1, superoxide dismutase transgenic mice on the FVB/N background. Differences between the groups were considered statistically significant when *p* < 0.05 (*), very significant when *p* < 0.01 (**), and extremely significant when *p* < 0.001 (***).

The data from functional measurements and immunohistochemical analysis suggested that the SOD1/GFAP/EGFP mice could suffer from potassium imbalance. Higher potassium levels are generally associated with pathological stages, however, to the best of our knowledge, the actual potassium concentration in the cerebrospinal fluid of SOD1/C57Bl6 or the SOD1/GFAP/EGFP mouse model is not known. We thus decided to use the elemental analysis and determine the concentration of extracellular potassium and other elements in the cerebrospinal fluid (CSF) of the 120 ± 3 day old SOD1/GFAP/EGFP mice experiencing stiffness and loss of ability to move their hind limbs together with ~20% weight loss as is typical for the final stage of the disease. We employed electrothermal vaporization (ETV) coupled with inductively coupled plasma optical emission spectrometry (ICP-OES) and measured samples of CSF collected from both CTRL and SOD1 mice. The CSF was isolated using protocol adapted from (Kaur et al., 2023), and only clear samples without any sign of blood (Figure 7A) were processed and analyzed. In addition to potassium, we also analyzed Ca, Fe, Mg, Na, P, and S (Table 2). The potassium concentration in the CSF from SOD1 mice was comparable to the concentration in CTRLs (Figure 7B) and both were within the range of the physiological concentration, which can slightly vary depending on factors such as age or experimental condition, but is typically ~3 mmoL/L (Larsen et al., 2016). Interestingly, we detected a significantly lower concentration of magnesium in the CSF of SOD1 mice (Figure 7C). Magnesium is among other things involved in muscle function and neuronal signaling, and a lower magnesium concentration has been reported in *post mortem* analysis of ALS patients as well (Yasui et al., 1997). Concentrations of other tested elements did not differ between the controls and mutated mice.

**Table 2:** Concentration of elements in the CSF of GFAP/EGFP mice.

mM/l	SOD1	CTRL
Ca	1.12 ± 0.1	1.25 ± 0.05
Fe	0.01 ± 0.01	0.01 ± 0.01
K	3.20 ± 0.21	3.49 ± 0.26
Mg	0.76 ± 0.03	0.81 ± 0.05
Na	152.20 ± 2.53	149.73 ± 7.64
P	1.38 ± 0.24	1.45 ± 0.14
S	0.68 ± 0.25	0.83 ± 0.2

SOD1, superoxide dismutase transgenic mice on the FVB/N background; CTRL, control mice on the FVB/N background.

## 4 Discussion

Amyotrophic lateral sclerosis is a fatal neurological disease with rapid progression and no effective treatment strategies. Even though it is primarily viewed as a motor-neuron disease, glial cells actively contribute to the onset/progression, and therefore represent promising targets for therapeutic approaches in the future. Astrocytes are considered especially promising, due to their crucial role in maintaining neural function and sustaining homeostasis of ions and transmitters. Nevertheless, it is necessary to understand the complex processes that are affected by the ALS pathology first, in order to identify optimal therapeutic strategies.

In this study, we detailed a mouse strain, created by crossbreeding the GFAP/EGFP (Nolte et al., 2001) with the SOD1(G93A) model of ALS (Gurney et al., 1994), resulting in mice with ALS-like phenotype and fluorescently labeled astrocytes. The original SOD1(G93A) model is based on a C57Bl6 background, while the GFAP/EGFP mice have FVB/N background. By extensive breeding we obtained a SOD1 mouse on FVB/N background (see Methods). This mouse just recently became commercially available for experimental use,^3^ however, without the labeled astrocytes. Behavioral testing of our mice revealed significant differences between disease progression of the original model and the one with the FVB/N background. The researchers providing this model to the Jackson Laboratory also highlighted this. It is very likely the different genetic background causing the discrepancies; however, the exact mechanisms through which it affects the disease progression are unclear. Previous studies on this topic revealed that various ALS models have phenotype variations affecting the progression. It can be, for instance, a variation in microglia–mediated neuroinflammation (Heiman-Patterson et al., 2011; Nikodemova and Watters, 2011) and/or the hyperactivity of metabolic processes. Jackson laboratories in particular report hyperactivity of metabolism in terms of increased oxygen consumption, abnormal energy expenditure or decreased circulating insulin levels for the B6SJL background.^4^ The different backgrounds affected the performance on both the hanging wire grid and Rotarod, but especially the motor coordination tested by Rotarod seemed to be much more affected in the SOD1/GFAP/EGFP model than in the SOD1/C57Bl6. Based on the gross phenotype, the progression is more rapid, nevertheless the mice still exhibit the distinctive features typical for ALS and represent thus another valid model for ALS research.

Our data from the fluorescence analysis of astrocytes confirmed the ALS-like pathology, also on the cellular level. We identified astrogliosis in both the ventral horns of the spinal cord and in the motor and somatosensory cortex, which confirms that astrocytes become activated during ALS, change their morphology, and may alter their functions. The astrocytic activation was observed in the spinal cord of the original SOD1/C57Bl6 model (Nagai et al., 2007; Baker et al., 2015), however, cortical astrocytes are a subject of debate. Some studies confirmed their activation in the motor cortex of SOD1 mice (Miller et al., 2018; Gomes et al., 2019; Gomes et al., 2020), but others (Filipi et al., 2023; Niessen et al., 2006) did not report any pathological changes, suggesting the pathology may be limited to the spinal and bulbar motor neurons in this particular model. To the best of our knowledge, no studies have assessed the astrocytic activation in the SOD1 model on FVB/N background yet, and we thus cannot compare our findings. Nevertheless, it is likely that with the faster progression rate of the gross phenotype, the cellular phenotype could also worsen/be easier to detect in comparison with the original SOD1/C57Bl6.

Besides morphology, astrocytes significantly change their gene profile in ALS (Baker et al., 2015; Miller et al., 2018; Ferraiuolo et al., 2011), especially in association with alterations of their two essential functions – glutamate uptake and potassium clearance. To broaden the knowledge of the latter, we took advantage of fluorescently labeled astrocytes and measured their swelling, reflecting their ability of K^+^ uptake and changes in the extracellular space during exposure to high K^+^ concentrations. K^+^ physiological concentration in the nervous system is ~3 mM and rises during pathological conditions such as ischemic stroke, traumatic brain injury, and epileptic seizures (Ohno et al., 2021). It happens primarily due to cellular damage, ion pump dysfunctions, excitotoxicity, or even blood–brain barrier dysfunction (Janigro, 2012; Keep et al., 1999; Lapilover et al., 2012; David et al., 2009; Larsen et al., 2016). The disruption of potassium homeostasis can affect neuronal activity and lead to further damage and dysfunction if not properly regulated.

Altered astrocytic ability of maintaining K^+^ homeostasis has been previously proposed in neurodegenerative disorders such as Huntington disease and Alzheimer’s disease as well as in ALS (Ding et al., 2024; Khakh et al., 2017; Ohno et al., 2021). Ding et al. (2024) and Stevenson et al. (2023) recently observed a reduced K^+^ clearance rate in the motor cortex of the SOD1/C57Bl6 mouse model, and those changes correlated with motor neuron loss (Ding et al., 2024). Our data however, showed a comparable degree of swelling in both CTRL and SOD1 cortical astrocytes, suggesting that the K^+^ uptake remains unimpaired in the cortex of the SOD1/GFAP/EGFP mice. The uptake is however, only one of the complex clearance mechanisms maintaining the K^+^ homeostasis. A possible interpretation for the divergence in our data has been proposed by Stevenson et al. (2023), who explain the reduced clearance by reduced coupling of astrocytes that prevent spatial buffering rather than by a direct change in potassium channel expression, hence the K^+^ uptake itself. The SOD1 spinal astrocytes, on the other hand, swelled significantly less than CTRL astrocytes during the highly elevated (50 mM) potassium concentration and volume recovery was appropriately faster. This suggests that swelling in our case reflects a change in the uptake mechanism, not a mechanism resulting in volume regulation. We hypothesize that this weaker reaction of SOD1 astrocytes can result from the altered mechanisms of astrocyte swelling, for example due to a loss or dysfunction of potassium channels or ion co-transporters such as NKCC1. The key channels in the removal of K^+^ from the extracellular space are Kir4.1, which are expressed exclusively in glial cells and primarily in astrocytes. Their altered expression has been previously described in rat (Bataveljic et al., 2012) and mouse (Kaiser et al., 2006) models of ALS with SOD1(G93A) mutation. Consistent with our results, lower expression of Kir4.1 was shown in the spinal cord (Kaiser et al., 2006) but not in the cerebral cortex (Bataveljic et al., 2012; Stevenson et al., 2023). Finally, the effect of increased K^+^ concentration on the activity of other transport mechanisms, such as glutamate transporters and others, should be mentioned. Since increased extracellular K^+^ concentration induces depolarization of astrocyte membranes (Du et al., 2018), other cellular voltage-dependent processes are likely to be affected. Therefore, we cannot exclude that the reduced astrocyte swelling observed under exposure to high K^+^ levels is not only due to changes in Kir4.1 expression, but also reflects changes in the expression/function of glutamate or ion co-transporters.

Astrogliosis and the volume regulation impairment we observed are closely connected to changes in the ECS diffusion parameters. Results from RTI measurements performed in the brain and spinal cord slices unveiled alterations in the ECS parameters following the exposure to two distinct potassium concentrations – 20 and 50 mM K^+^. Application of both K^+^ concentrations resulted in a noted decrease in the ECS volume fraction, indicating a compensatory shrinkage of the ECS caused by swelling of cells and/or their components. Our measurements revealed that the effect of lower potassium concentration (20 mM) does not show any significant differences in a typical time course of the ECS parameter changes between the CTRL and SOD1 mice in either the brain or the spinal cord.

In the cortex, the inter-group differences were also not evoked with the higher concentration of potassium (50 mM). In contrast, the measurements in the spinal cord indicated that 50 mM K^+^ has a more pronounced effect in CTRL than in SOD1 mice – a drop of 32% (CTRL) and 13% (SOD1) from the initial values of the ECS volume fraction and a significant increase in tortuosity in CTRL but not in SOD1 mice. Regarding tortuosity, it is worth mentioning that its values in the spinal cord can be affected by the presence and density of the myelinated fibers that represent a major source of the diffusion barriers (Thorne et al., 2005) and influence even the initial tortuosity values, which were higher in the spinal cord than in the brain. The increase of tortuosity in the spinal cord of the CTRL mice can be therefore attributed at least partially to the closer alignment of myelin fibers, due to cellular swelling and the subsequent reduction in extracellular space that was larger in CTRL than in SOD1 mice. Our previous studies revealed that both ECS volume fraction and/or tortuosity could be strongly affected by the dynamic changes of the extracellular matrix (ECM) (Zamecnik et al., 2012; Zamecnik et al., 2004; Sucha et al., 2020). Alterations in the ECM composition were certainly shown in SOD1 rats in several studies, where they reported abnormally organized or damaged protective perineuronal net structures around the spinal MNs, as well as different expression profiles of chondroitin sulfate proteoglycans (CSPGs) (Forostyak et al., 2020; Forostyak et al., 2014; Cheung et al., 2024). We could thus expect that ECM alterations in the SOD1 tissue may affect the initial values of the ECS diffusion parameters. However, this assumption was not confirmed by our study as we did not detect any significant differences in the initial values of *α* and *λ* between CTRL and SOD1 mice either in the brain or spinal cord. It is not very likely that slowly developing and prolonged changes in the ECM composition might affect the rapidly developing ECS diffusion changes during acute cell swelling. We can only speculate that changes in the content of ECM, rich in water molecules bound to its negative charges, may affect the possibility of the cellular expansion and thus indirectly modulate cell swelling.

Comparison of the RTI measurements and astrocytic volume regulation analyses revealed similar responses, resulting from astrocyte swelling and compensatory shrinkage of the ECS volume in reaction to increased potassium concentrations and similar recovery during the washout period. Both techniques detected significant differences between CTRL and SOD1 mice only in the spinal cords during 50 mM K^+^. As demonstrated in our previous studies, such agreement of RTI and 3-D morphometry results is quite exceptional since the RTI-detected changes reflect the volume alterations of all cell types, not only those of the astrocytes (Kolenicova et al., 2020; Tureckova et al., 2021). Our findings therefore emphasize the crucial role of astrocytes in hyperkalemia-evoked cell swelling. This aligns with a study of Walch et al. (2020) showing that glial cells, particularly astrocytes, display marked vulnerability to alterations induced by potassium, while neurons show resistance to such disruptions. Walch et al. (2020) demonstrated that elevated extracellular potassium concentrations induce rapid and significant swelling of astrocytes, underscoring their crucial role in responding to changes in potassium levels. While astrocytes swiftly returned to baseline volume upon reverting to a control solution, they exhibited similar swelling responses upon reintroduction of elevated potassium levels. Conversely, neuronal volume remained largely unaffected by these potassium-induced changes, emphasizing the unique sensitivity of astrocytes to fluctuations in extracellular potassium and their pivotal role in maintaining cellular homeostasis (Walch et al., 2020).

As mentioned previously, the potassium homeostasis can be disrupted during pathological stages; however, it is not just potassium levels that can be altered during ALS. An imbalance of other ions such as Ca^2+^, Na^+^, or Mg^2+^ is considered a significant aspect of the ALS pathology. Previous studies for instance reported reduced expression of calcium-buffering in post-mortem isolated MNs in ALS patients (Alexianu et al., 1994), an increase in persistent sodium currents in spinal and cortical MNs in SOD1 mice (Pieri et al., 2009; Kuo et al., 2005; Kuo et al., 2004), and alterations of the fast transient sodium currents generated by Na_v_ channels in spinal MNs in SOD1 mice (Zona et al., 2006). Nevertheless, the precise measurement of these elements in animal models or patients is not very frequent. Our data from the analysis of the CSF showed similar concentrations of K^+^, Ca^2+^, Na^+^, Fe^2+^, P, and S in CTRLs and SOD1 samples. Potassium levels were previously determined in ALS SOD1/C57Bl6 mice by Ding et al. (2024). Using *in vivo* recording with K^+^-sensitive electrodes, they reported a significantly increased level of K^+^ in the cortex of diseased mice. We did not observe such a difference in the CSF of SOD1/GFAP/EGFP mice, which is most likely due to the different methodological approach. As Stevenson et al. (2023) showed, the rate of potassium clearance and concentration can differ even within parts of the cortex. Therefore, analysis of CSF would not detect any region-specific potassium level alterations. When discussing potassium clearance in the context of ALS, it is also worth mentioning oligodendrocytes, which contribute to the clearance of excessive K^+^. Similar to astrocytes, though, the Kir4.1 channel that is largely responsible for the clearance (Schirmer et al., 2018) was reported to be downregulated in the spinal cord of SOD1(G93A) rats (Peric et al., 2021). Interestingly, we detected a significantly decreased concentration of magnesium in CSF of ALS mice. Magnesium is involved in muscle function, the cellular antioxidant mechanism and neuronal signaling as it inhibits NMDA receptors, which helps to prevent excitotoxicity (Shindo et al., 2020). Thus, our data are in agreement with (Yasui et al., 1997), who found lower magnesium content in bones and ligaments in *post mortem* samples of ALS patients. Generally, magnesium deficiency has been considered a possible risk factor for ALS, which translated in further research when trying to explore the possibilities of magnesium supplementation to modify the course of the disease. However, at least in the SOD1 model, a study from Pamphlett et al. (2003) reported no increased survival or delayed disease onset of mice being supplemented by higher doses of magnesium.

## 5 Conclusion

For the purposes of this study, we generated a crossbred mouse model of ALS in which EGFP is expressed specifically in astrocytes. This model allowed us to perform a functional study to assess the capability of astrocytes to regulate extracellular ion concentrations. We confirmed that astrocytes adopt reactive morphology in the motor cortex and ventral horns of the spinal cord during ALS-like pathology in the SOD1/GFAP/EGFP model. Our findings from functional measurements suggest a spinal cord-specific alteration in astrocytic ability to take up potassium ions during exposure to high potassium concentrations, which is associated with a decrease in Kir4.1 expression. In cortical astrocytes, the ability of potassium uptake appears to remain unchanged and the expression of Kir4.1 is unaffected despite reactive morphology. These findings are manifested both at the level of astrocyte swelling and changes in ECS parameters. The observed differences in astrocyte buffering capacity highlight the complex interplay between regional astrocyte function and ALS pathophysiology, emphasizing the need for further investigations into region-specific mechanisms underlying potassium dysregulation in ALS.

## Data Availability

The raw data supporting the conclusions of this article will be made available by the authors, without undue reservation.

## Footnotes

^1^https://fiji.sc

^2^https://www.graphpad.com

^3^https://www.jax.org/strain/013199

^4^https://www.jax.org/strain/002726
